# Blood angiopoietin-2 predicts liver angiogenesis and fibrosis in hepatitis C patients

**DOI:** 10.1186/s12876-021-01633-8

**Published:** 2021-02-08

**Authors:** Yosuke Osawa, Sachiyo Yoshio, Yoshihiko Aoki, Masaaki Korenaga, Masatoshi Imamura, Takashi Oide, Miku Okawara, Hironari Kawai, Yuriko Tsutsui, Yuichi Yoshida, Shiori Yoshikawa, Taizo Mori, Taiji Yamazoe, Tatsuya Kanto

**Affiliations:** 1 The Research Center for Hepatitis and Immunology, National Center for Global Health and Medicine, 1-7-1 Kohnodai, Ichikawa, Chiba 272-8516 Japan; 2 Department of Gastroenterology, International University of Health and Welfare Hospital, 537-3 Iguchi, Nasushiobara, Tochigi 239-2763 Japan; 3 Department of Gastroenterology and Hepatology, Kohnodai Hospital, National Center for Global Health and Medicine, 1-7-1 Kohnodai, Ichikawa, Chiba 272-8516 Japan; 4 Department of Pathology and Laboratory Medicine, Kohnodai Hospital, National Center for Global Health and Medicine, 1-7-1 Kohnodai, Ichikawa, Chiba 272-8516 Japan

**Keywords:** Angiogenesis, Angiopoietin-2, Carbon tetrachloride, Chronic hepatitis C, Endothelin-1, Liver fibrosis

## Abstract

**Background:**

Pathological angiogenesis is involved in the development of hepatocellular carcinoma. In patients with chronic hepatitis C (CHC), the level of angiogenic factor angiopoietin (ANGP)-2 is reported to be increased in the blood, correlating with fibrosis. In this study, we aimed to clarify whether blood ANGP-2 is useful as a biomarker for liver angiogenesis and fibrosis in CHC patients and to further reveal the relationship between such pathology in a carbon tetrachloride (CCl_4_)-treated liver fibrosis mouse model.

**Methods:**

Plasma levels of ANGP-2, expression of a liver sinusoidal endothelial cell (LSEC) marker (CD31), collagen deposition (Sirius Red staining) in the liver, clinical fibrosis markers (Mac-2 binding protein glycosylation isomer, virtual touch quantification, and liver stiffness measurement), and liver function (albumin bilirubin score) were examined in CHC patients. To determine the effects of an anti-angiogenic agent on liver fibrosis in vivo, sorafenib was administered to the CCl_4_-treated mice (BALB/c male).

**Results:**

The plasma levels of ANGP-2 were increased in CHC patients compared to healthy volunteers and decreased by the eradication of hepatitis C with direct-acting antivirals. In addition, plasma ANGP-2 levels were correlated with CD31 expression, collagen deposition, clinical fibrosis markers, and liver function. Sorafenib inhibited liver angiogenesis and fibrosis in the CCl_4_-treated mice and was accompanied by decreased ANGP-2 expression in LSECs.

**Conclusions:**

ANGP-2 may serve as a useful biomarker for liver angiogenesis and fibrosis in CHC patients. In addition, angiogenesis and fibrosis may be closely related.

## Background

Chronic liver injury leads to the progression of liver fibrosis regardless of etiology, eventually leading to liver cirrhosis and hepatocellular carcinoma (HCC). Pathological angiogenesis and endothelial dysfunction are major drivers of tumor dissemination in the liver. Angiogenesis has been considered as a crucial pathophysiological mechanism for liver fibrosis because anti-angiogenic therapy has been shown to exhibit anti-fibrotic effects in some experimental models [1–3]. Sorafenib is a molecular-targeted drug for HCC that inhibits angiogenesis through receptors for vascular endothelial growth factor (VEGF) or platelet-derived growth factor and by suppressing tumor cell proliferation through the Raf-mediated pathway and receptor tyrosine kinases [4]. In addition, anti-fibrotic properties of sorafenib have been reported [5]. The VEGF and angiopoietin (ANGP)/Tie system are key regulators of angiogenesis in cancer tissues [6, 7]. In contrast to the contribution of ANGP-1 to vascular stability, ANGP-2 stimulates its remodeling and destabilization [8–10], resulting in angiogenesis. Endothelin-1 (ET-1), another angiogenic factor, has also been reported to be involved in liver fibrosis [11]. Therefore, it is likely that VEGF, ANGP-2, and ET-1 play active roles in supporting the relationship between angiogenesis and fibrosis in chronic liver disease.

The hepatitis C virus (HCV) is a major cause of chronic liver disease-related deaths due to liver cirrhosis and HCC worldwide. As a result of the development of potent direct anti-viral agents (DAAs), the prevalence of chronic HCV carriers and mortality from HCV-induced HCC have declined in recent years. Several reports have been published regarding the impact of ANGP-2 on the pathogenesis of chronic HCV infection. Serum ANGP-2 levels have been observed to be significantly higher in patients with chronic hepatitis C (CHC) than in healthy controls while VEGF levels were comparable between the two groups [12, 13]. Serum ANGP-2 levels appear to be correlated with liver stiffness in CHC patients [14] and increase based on stage progression from chronic hepatitis to cirrhosis [15]. In patients with decompensated cirrhosis, serum ANGP-2 may be a predictor of mortality [16]. In CHC patients who later developed HCC, ANGP-2 liver expression levels were higher than in patients who did not develop HCC [17]. These reports suggest that ANGP-2 is one of the crucial factors that link angiogenesis with fibrosis as a reflection of the risk potential for liver carcinogenesis.

In this study, we aimed to clarify whether plasma ANGP-2 is useful as a biomarker for both liver angiogenesis and fibrosis in patients with CHC. To further elucidate the relationship between angiogenesis and fibrosis in vivo, we examined the impact of ANGP-2 on liver pathology using sorafenib in a carbon tetrachloride (CCl_4_)-induced liver fibrosis mouse model.

## Methods

### Patients

Blood samples (n = 20) were obtained from CHC patients who were taking DAAs. All patients achieved a sustained viral response at 12 weeks (SVR12), and blood samples were again collected at 12 weeks after the treatment. Liver biopsy specimens were obtained from 14 of the 20 patients. Clinical data were obtained from their electronic clinical records. For healthy controls, blood samples (n = 15) were donated from healthy volunteers (HV) who served as hospital officers and did not display any abnormalities in their annual health check, including blood examination. The liver stiffness measurement (LSM) was performed using a FibroScan 502 (Echosens, Waltham, MA, USA), and Virtual Touch Quantification (VTQ) by acoustic radiation force impulse was performed using an ACUSON S3000 ultrasound system (Siemens, Erlangen, Germany).

### Animals and treatments

Male wild-type BALB/c mice (wild-type) aged 8 weeks were obtained from Japan SLC (Shizuoka, Japan). The mice were bred in specific pathogen free condition (12 h light/dark cycle, free access to water (automatic water supply) and food (CE-2, CLEA Japan, Tokyo, Japan)). The animals were intraperitoneally injected with 1 mL/kg body weight CCl_4_ (1:10 v/v in corn oil) (Sigma-Aldrich, St. Louis, MO, USA) twice a week for 2 weeks from 9 weeks of age in home cages (n = 4). In addition, the animals were administered 750 μg/mouse sorafenib tosylate (CS-0164; Chem Scene, Monmouth Junction, NJ, USA) dissolved in 200 μL 0.5% carboxylmethylcellulose via oral gavage 3 times a week for 2 weeks as needed (n = 4). Control animals were intraperitoneally administrated with corn oil (n = 4). After confirmation of unconsciousness by anesthesia with intraperitoneal injection with medetomidine (0.75 mg/kg), midazolam (4 mg/kg), and butorphanol tartrate (5 mg/kg), the animals were euthanized 3 days after the final CCl_4_ treatment by exsanguination from the inferior vena cava using 25-gauge needle and syringe. The liver was immediately removed, and a section of the dissected tissue was frozen in liquid nitrogen. A part of the tissues was fixed with 10% formalin or embedded in optimal cutting temperature compound (Sakura Finetek Japan, Tokyo, Japan) for histological analysis. Based on our previous data that hydroxyproline is increased by CCl_4_ and that sorafenib reduces liver fibrosis in other mouse models, the sample size was determined (n = 4 in each group). The animals were randomly allocated to the groups. The experiment was performed as a single set and all animals were analyzed.

### Enzyme-linked immunosorbent assay (ELISA)

ANGP-2 and ET-1 levels in the plasma were determined by ELISA (Human Angiopoietin-2 and Endothelin-1 Quantikine ELISA kits; R&D Systems, Minneapolis, MN, USA).

### Histological analysis

The liver was fixed with 10% formalin, paraffine embedded, sectioned, and stained with hematoxylin and eosin (H&E). Collagen deposition was stained with Sirius Red (saturated picric acid containing 0.1% DirectRed 80 and 0.1% FastGreen FCF; Sigma-Aldrich). CD31 was stained in human samples with the anti-CD31 antibody (11265-1-AP; Proteintech, Rosemont, IL, USA) and EnVision + Dual Link System (DAKO, Carpinteria, CA, USA). Diaminobenzidine tetrahydrochloride was used as the peroxidase substrate, and the sections were counterstained with hematoxylin. For immunostaining of CD31, CD146, and ANGP-2, 5-μm-thick frozen liver sections were cut on a cryostat, fixed with methanol/acetone, and stained with the following specific antibodies: fluorescein isothiocyanate (FITC)-conjugated CD31 (11-0311-81; Invitrogen, Carlsbad, CA, USA), phycoerythrin (PE)-conjugated CD146 (134704; BioLegend, San Diego, CA, USA), and ANGP-2 (ab155106; Abcam, Cambridge, UK). Alexa Fluor 546-conjugated anti-rabbit IgG (A11035; Invitrogen) was used as the secondary antibody. The nuclei were stained with mithramycin and 4′-6-diamindino-2-ohenylindole. The Sirius Red-positive area or CD31-positive area of the liver specimens was quantified using ImageJ software (National Institutes of Health, Bethesda, MD, USA) and shown as a percentage of the total section area. The specimens were observed using a microscope (Z9000; Keyence, Osaka, Japan).

### Hydroxyproline measurement

Liver tissue was homogenized and hydrolyzed for 24 h at 110 °C in 6 N HCl. The samples were oxidized with chloramine-T (Sigma-Aldrich) and incubated in Ehrlich’s perchloric acid solution. The sample absorbance was measured at 558 nm. Purified hydroxyproline (Sigma-Aldrich) was used as the standard. Hydroxyproline content was expressed as µg hydroxyproline/g liver.

### Real-time quantitative reverse transcription polymerase chain reaction (qRT-PCR)

A High-Capacity cDNA Reverse Transcription Kit (Applied Biosystems, Foster City, CA, USA) was used for reverse transcription. Real-time qRT-PCR was performed using SYBR Premix Ex Taq (Takara, Shiga, Japan) for ANGP-2 (TCCAAGAGCTCGGTTGCTAT and AGTTGGGGAAGGTCAGTGTG), CD146 (CCCAAACTGGTGTGCGTCTT and GGAAAATCAGTATCTGCCTCTCC), ANGP-1 (TGCAGCAACCAGCGCCGAAA and CAGGGCAGTTCCCGTCGTGT), VEGFA (AAAGGCTTCAGTGTGGTCTGAGAG and GGTTGGAACCGGCATCTTTATC), and ET-1 (TTCCCGTGATCTTCTCTCTGC and CTGCACTCCATTCTCAGCTCC) as well as the probe and primer sets (Applied Biosystems) for 18S rRNA (Hs99999901s1) with the Thunderbird Probe qPCR mix (Toyobo, Tokyo, Japan). PCR was performed on a LightCycler 480 (Roche Applied Science, Mannheim, Germany). The measured changes were normalized based on 18S rRNA values.

### Alanine aminotransaminase (ALT) and aspartate aminotransferase (AST) measurements

Plasma ALT and AST levels of the animals were measured using SPOTCHEM D (Arkray, Kyoto, Japan).

### Statistical analysis

The results are expressed as the means ± standard deviations (SDs) of data collected from at least three independent experiments. The data were compared between groups using a one-way analysis of variance (ANOVA), the Kruskal–Wallis test, or the two-tailed Student’s *t*-test. Pearson’s or Spearman’s rank correlation coefficients were used for correlation analysis. *P* < 0.05 was considered statistically significant. Statistical analyses were performed using GraphPad Prism software version 5 (GraphPad Software Inc., San Diego, CA, USA).

## Results

### Blood ANGP-2 level associations with liver fibrosis and liver function in CHC patients

**Fig. 1:**
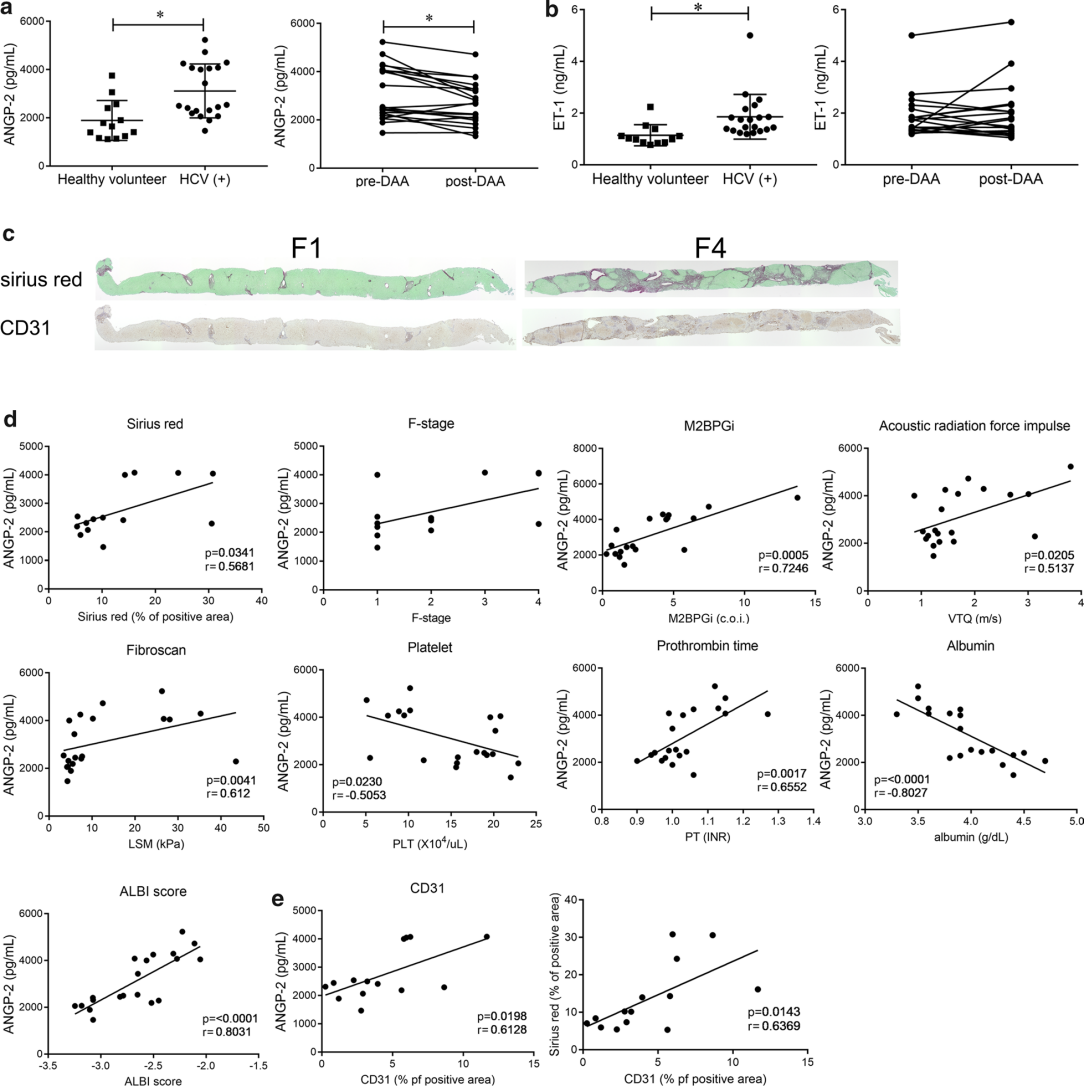
ANGP-2 expression in the plasma of CHC patients. **a**, **b** Plasma ANGP-2 and ET-1 levels in CHC patients (HCV (+)) were determined using ELISA and compared to those in healthy volunteers (HV) (left panels). The effects of eradication were examined (right panels). **c** Collagen deposition was assessed by Sirius Red staining. CD31 expression in the liver was examined by immunohistochemistry (original magnification: × 40). **d** Correlation of the plasma ANGP-2 level with the Sirius Red-positive area in the liver, F-stage, serum M2BPGi, VTQ, LSM, platelet count, prothrombin time, serum albumin, and ALBI score are shown. **e** Correlation of the CD31-positive area in the liver with the plasma ANGP-2 level and Sirius Red-positive area are shown. The results are provided as means ± SDs. *P < 0.05 based on a two-tailed Student’s *t*-test. The values for Spearman’s or Pearson’s correlation coefficient are indicated

Twenty CHC patients were included in this study. The clinical characteristics of the patients are summarized in Table 1. The plasma levels of ANGP-2 were increased in the CHC patients compared to the HV group (Fig. 1a, left panel). All 20 patients achieved SVR12, and the levels of ANGP-2 were reduced at SVR12 (Fig. 1a, right panel). Similarly, ET-1 level was elevated in CHC patients, but it did not change after HCV eradication (Fig. 1b). We examined the relationships between blood ANGP-2, angiogenesis, and fibrosis in 14 patients whose blood samples and liver specimens were obtained before DAA therapy was applied. In liver specimens diagnosed as stage 4 fibrosis (F4), the Sirius Red-positive area indicating collagen deposition and CD31 expression, a marker for capillarized LSECs, were increased compared to those in F1 specimens (Fig. 1c and Additional file 1: Fig. 1a). Plasma ANGP-2 levels had a positive correlation with the Sirius Red-positive area and also appeared to correlate with the Metavir F-stage score (Fig. 1d). In addition, ANGP-2 was positively correlated with Mac-2 binding protein glycosylation isomer (M2BPGi), VTQ, LSM, prothrombin time, and albumin bilirubin (ALBI) score but negatively correlated with platelet and albumin levels (Fig. 1d). These results show that blood ANGP-2 reflects the degree of liver fibrosis and liver function. ANGP-2 also showed a positive correlation with the CD31-positive area (Fig. 1e). Furthermore, a positive correlation was observed between CD31 and Sirius Red-positive areas (Fig. 1e). Therefore, ANGP-2, angiogenesis, and fibrosis were mutually related in patients with CHC. In contrast, ALT, α-fetoprotein (AFP), and des-γ-carboxy prothrombin (DCP) had no correlation with ANGP-2 (Additional file 1: Fig. 1b). ET-1 levels did not show any correlations with the Sirius Red-positive area or ALT (Additional file 1: Fig. 1a). To examine the value of ANGP-2 for prediction of liver fibrosis, the performance of ANGP-2 was compared with the well-known fibrosis prediction indices, FIB-4 index and AST to platelet ratio index (APRI), and LSM (Additional file 1: Fig. 1c). ANGP-2 levels discriminated F-stage 2–4 patients from F-stage 1 patients with an area under the curve (AUC) of 0.729. LSM showed superior predictive ability and the performance of ANGP-2 was not inferior to those of FIB-4 and APRI.

**Table 1:** Blood ANGP-2 measurement profiles of the study participants. The results are provided as means ± SDs

	Healthy volunteers (HVs)	Hepatitis C virus ( +) (HCV) patients
Number	15	20
Age (median, range)	26 (21–47)	70 (38–84)
Gender (M/F)	2/13	11/9
Glecaprevir + Pibrentasvir	–	14
Ledipasvir + Sofosbuvir	–	5
Elbasvir + Grazopervir	–	1
ANGP-2 (pg/mL)	1896 ± 771.7	3115 ± 1116
M2BPGi (c. o. i.)	N. A	3.56 ± 3.25
VTQ (m/s)	N. A	1.74 ± 0.80
PLT (× 10^4^/μL)	N. A	14.9 ± 5.7
ALBI score	N. A	– 2.667 ± 0.3693
PT (INR)	N. A	1.04 ± 0.09
Albumin	N. A	4 ± 0.4
ALT (IU/L)	N. A	48.80 ± 37.65
AFP (ng/mL)	N. A	15.4 ± 34.2
DCP (mAU/mL)	N. A	20.2 ± 9.5
Pathological examination	N. A	14
Metavir F-stage F0/F1/F2/F3/F4		0/6/4/1/3

ANGP-2; angiopoietin-2; VTQ, virtual touch quantification; PLT, platelet; ALBI score, albumin bilirubin score; PT, prothrombin time; ALT, alanine transaminase; AFP, alpha-fetoprotein; DCP, des-γ-carboxy prothrombin

### Sorafenib-mediated reductions of angiogenesis and fibrosis accompanied by decreased ANGP-2 expression in CCl_4_-treated mice

**Fig. 2:**
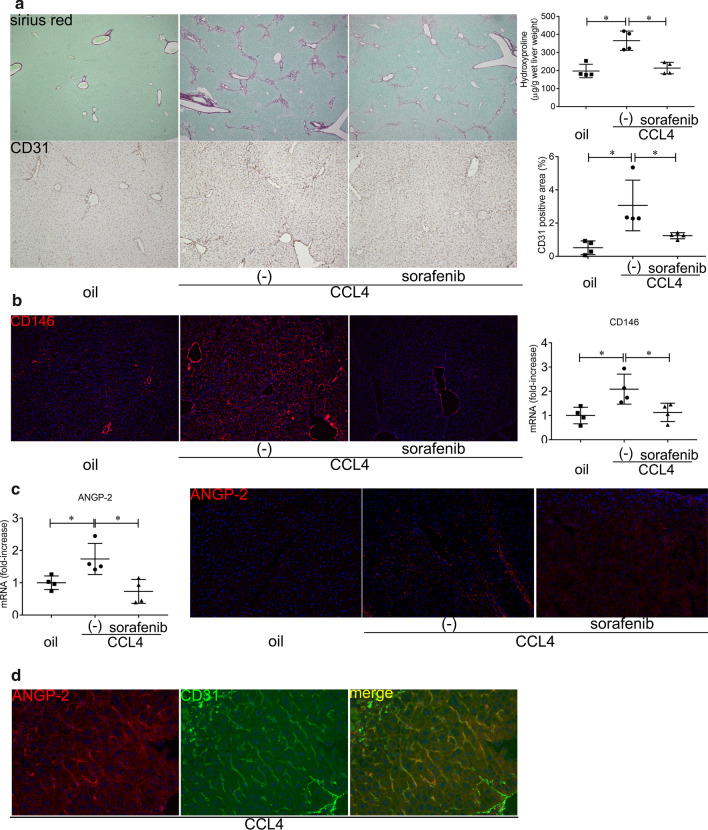
ANGP-2 expression in mouse liver fibrosis induced by CCl_4_. BALB/c male mice were treated with CCl_4_ (1 mL/kg twice a week) with or without sorafenib (750 μg 3 times a week) for 2 weeks and humanely sacrificed. **a** Collagen deposition was assessed by Sirius Red staining (original magnification: × 40) and hydroxyproline content in the liver (upper panels). CD31 expression in the liver was examined by immunohistochemistry (original magnification: × 100), and the CD31-positive area (%) was quantified (lower panels). **b** CD146 expression in the liver was examined by immunohistochemistry using the anti-PE-conjugated CD146 antibody (original magnification: × 100) (left panels). CD146 expression in the liver was determined by real-time qRT-PCR (right panel). **c** ANGP-2 mRNA expression in the liver was determined by real-time qRT-PCR (left panel). ANGP-2 was stained with a primary antibody and an Alexa Fluor 546-conjugated secondary antibody (original magnification: × 200) in the liver (right panels). **d** ANGP-2 and CD31 in the liver were double stained with the following antibodies: anti-ANGP-2 antibody, Alexa Fluor 546-conjugated secondary antibody, and FITC-conjugated anti-CD31 antibody (original magnification: × 400). The two images were merged to demonstrate ANGP-2 expression in LSECs. The results are representative of at least four independent experiments and provided as means ± SDs. *P < 0.05 based on a one-way ANOVA. The values for Spearman’s or Pearson’s correlation coefficient are indicated

Treatment with CCl_4_ is a well-known stimulus to induce liver fibrosis in mice. CCl_4_ administration increased the Sirius Red-positive area and hydroxyproline accompanied by higher CD31 and CD146 expression levels in the liver, indicating that fibrosis and angiogenesis occurred in the mice (Fig. 2a, b). To examine the effects of sorafenib on angiogenesis and liver fibrosis, the CCl_4_-treated mice were administered sorafenib. Significant decreases were observed in the Sirius Red-positive area as well as hydroxyproline, CD31, and CD146 expression levels in the mice treated with sorafenib (Fig. 2a, b). Thus, sorafenib inhibited angiogenesis and liver fibrosis in the CCl_4_-treated mice. In contrast, sorafenib did not reduce ALT levels (Additional file 2: Fig. 2a), suggesting that the anti-fibrotic effects of sorafenib are not due to reductions in hepatocellular damage and inflammation.

ANGP-2 expression increased in the livers of mice treated with CCl_4_, and the induction was inhibited by sorafenib treatment (Fig. 2c). Immunofluorescence analysis revealed that ANGP-2 expression was co-localized with CD31 (Fig. 2d), indicating that LSECs are a source of ANGP-2. In the CCl_4_-treated mice, the mRNA levels of ANGP-1, VEGFA, and ET-1 were comparable to those in the control group, and sorafenib failed to affect their respective expression levels (Additional file 2: Fig. 2b).

## Discussion

In this study, we found that ANGP-2 levels were elevated in the blood of CHC patients. The levels of ANGP-2 reflected the degree of liver angiogenesis and fibrosis as well as represented liver functions in patients. Sorafenib reduced liver angiogenesis and fibrosis in CCl_4_-treated mice accompanied by a reduction in LSEC-derived ANGP-2. These findings show that ANGP-2 is a useful biomarker for liver angiogenesis and fibrosis in CHC patients and raises the possibility that angiogenesis and fibrosis are closely related.

Angiogenesis and fibrosis are crucial phenomena in the pathogenesis of chronic liver disease and hepatocarcinogenesis. However, it has yet to be determined whether angiogenesis occurs in parallel with the development of fibrosis or plays a causal role in fibrosis. Angiogenesis induces collagen fibril condensation that leads to mechanical force-mediated HSC activation [2]. The LSECs secrete angiogenic factors, including ANGP-2, which may further contribute to angiogenesis that results in the activation of HSCs and progression of liver fibrosis. LSECs lose their fenestrae and develop a basement membrane in a process known as capillarization and promote angiogenesis [18] resulting in the transformation of fenestrated hepatic sinusoids into continuous capillaries followed by increased CD31 expression. Capillarized LSECs lose their ability to prevent HSC activation [19]. In contrast, angiogenesis is regulated by AGNP-1 secreted by activated HSCs [20].

It has been reported that ANGP-2 blood levels are associated with liver fibrosis in CHC patients [14]. In this study, blood levels of ANGP-2 were correlated with CD31 expression and liver fibrosis. ANGP-2 was expressed in capillarized LSECs. Thus, ANGP-2 may reflect the status of angiogenesis rather than HSC activation. In support of this, serum ANGP-2 levels have been reported to correlate with hepatic angiogenesis in nonalcoholic steatohepatitis patients, and ANGP-2 inhibitors were observed to reduce angiogenesis and fibrosis in the fibrotic livers of mice fed a methionine- and choline-deficient diet [1]. After eradication of HCV from patients treated with DAAs, ANGP-2 decreased while ET-1 did not. In the mouse model, ANGP-2 was only expressed in LSECs. Thus, improvements in inflammation by DAAs may reduce the angiogenic response of LSECs, resulting in decreased ANGP-2 secretion. In contrast, ET-1 is released by activated HSCs other than LSECs [21], suggesting that ET-1 does not reflect the status of LSECs.

In this study, sorafenib inhibited angiogenesis in the liver of CCl_4_-treated mice. ANGP-1 stabilizes blood vessels in normal conditions. ANGP-2 induces destabilization by inhibition of ANGP-1, which facilitates vessel proliferation or sprouting in the presence of VEGF [8–10]. Sorafenib may have dual inhibitory effects: anti-VEGF effects by affecting the VEGF receptor and mediated signal in LSECs as well as anti-ANGP-2 effects by decreasing ANGP-2 production by LSECs. A combination of sorafenib and ANGP-2-targeted agents has been evaluated for the treatment of cancer in some clinical trials [10]. In HCC tissues, VEGF and ANGP-2 are secreted by cancer cells [22]. In contrast, ANGP-2 was expressed by capillarized LSECs in the fibrosis model, suggesting that ANGP-2 works in an autocrine manner. Indeed, ANGP-2 expression was reduced by sorafenib in our study. In contrast to ANGP-2, the expression levels of ET-1 and VEGF were not affected by sorafenib, suggesting that their production occurs in another source beyond LSECs [21, 23]. Sorafenib also directly affects HSCs, resulting in the inhibition of proliferation and collagen synthesis as well as induction of apoptosis and collagen degradation [24]. Thus, sorafenib is capable of inhibiting liver fibrosis through complex communications between LSECs and HSCs.

## Conclusions

Blood ANGP-2 levels are associated with liver angiogenesis, fibrosis, and liver function in patients with CHC. In a mouse model of fibrosis, sorafenib reduced liver angiogenesis and fibrosis accompanied by a decrease in LSEC-derived ANGP-2. Thus, ANGP-2 may serve as a potential biomarker and therapeutic target for mediating the sequence of angiogenesis and fibrosis in the liver.

## Supplementary Information

Additional file1.

Additional file2.

## Data Availability

All data generated or analyzed during this study are included in this published article and its supplementary information files.

## Abbreviations

HCC: Hepatocellular carcinoma
CHC: Chronic hepatitis C
ANGP: Angiopoietin
CCl_4_: Carbon tetrachloride
LSEC: Liver sinusoidal endothelial cell
VEGF: Vascular endothelial growth factor
ET-1: Endothelin-1
HCV: Hepatitis C virus
DAA: Anti-viral agent
SVR: Sustained viral response
HV: Healthy volunteers
LSM: Liver stiffness measurement
VTQ: Virtual Touch Quantification
M2BPGi: Mac-2 binding protein glycosylation isomer
ELISA: Enzyme-linked immunosorbent assay
H&E: Hematoxylin and eosin
FITC: Fluorescein isothiocyanate
PE: Phycoerythrin
qRT-PCR: Real-time quantitative reverse transcription polymerase chain reaction
ALT: Alanine aminotransaminase
AST: Aspartate aminotransferase
ANOVA: A one-way analysis of variance
AFP: α-Fetoprotein
DCP: Des-γ-carboxy prothrombin
FIB-4: FIB-4 index
APRI: Aspartate aminotransferase to platelet ratio index
AUC: Area under the curve
